# Gender-specific cardiac features in Andersen–Tawil syndrome: a comprehensive meta-analysis of case reports and series

**DOI:** 10.1007/s10840-026-02237-6

**Published:** 2026-01-14

**Authors:** Alan Garcia, Abdul Mueez Alam Kayani, Ricky Lemus-Zamora, Daniel Alejandro Navarro-Martinez, Eduardo Tellez-Garcia, Richard Salama-Frisbie, Jorge Gomez Flores, Eduardo Aviles, Brijesh Patel

**Affiliations:** 1 Department of Internal Medicine, Indiana University School of Medicine, 350 W. 14th Street, HA 7024, Indianapolis, IN 46202 USA; 2 Department of Internal Medicine, AdventHealth Tampa, Tampa, FL USA; 3 Department of Internal Medicine, Instituto Nacional de Nutrición Salvador Zubirán, Ciudad de México, México; 4 Department of Internal Medicine, Instituto Nacional de Cardiologia Ignacio Chavez, Ciudad de México, México; 5 Department of Cardiovascular Medicine, Indiana University School of Medicine, Indianapolis, IN USA

**Keywords:** Andersen-Tawil syndrome, Ventricular arrhythmia, Cardiac arrest, Sex-specific risk

## Abstract

**Background:**

Andersen-Tawil Syndrome (ATS) is an autosomal dominant disorder defined by a classic triad of dysmorphic features, episodic muscular weakness, and ventricular arrhythmias. Notably, gender appears to significantly influence clinical outcomes, with females constituting the predominant affected demographic and exhibiting poorer cardiovascular prognoses.

**Methods:**

This study seeks to outline the gender-specific clinical features of ATS, with an emphasis exclusively on patients exhibiting cardiac manifestations. A total of 114 case series and reports were collected from 1994 to 2024 from Scopus, Embase, PubMed, Web of Science, CINAHL, and Cochrane Library. Only patients exhibiting cardiovascular manifestations were included in the study, excluding cohorts without single patient characteristic descriptions. Analysis was performed using the available data for each variable.

**Results:**

The studied population comprised 259 cases, of whom 176 were female. The analysis demonstrated that females are less likely to present with the characteristic triad than males (*p* = 0.03). Additionally, female patients were less likely to present with periodic paralysis than males (*p* = 0.02). Overall, premature ventricular contractions (PVCs) were the most common arrhythmia, bigeminy being the most common presentation. Polymorphic PVCs were more prevalent in females than males (*p* = 0.01). Females experience more ventricular tachycardia episodes than males, especially bidirectional (*p* = 0.01). Thirty-seven patients had an episode of cardiac arrest, either fatal or non-fatal. Females were more prone to develop sudden cardiac arrest compared to males (*p* = 0.02).

**Conclusion:**

ATS females with cardiac manifestations may require closer follow-up due to the higher risk of developing life-threatening arrhythmias and cardiac arrest than males.

**Graphical Abstract:**

**Figure:**
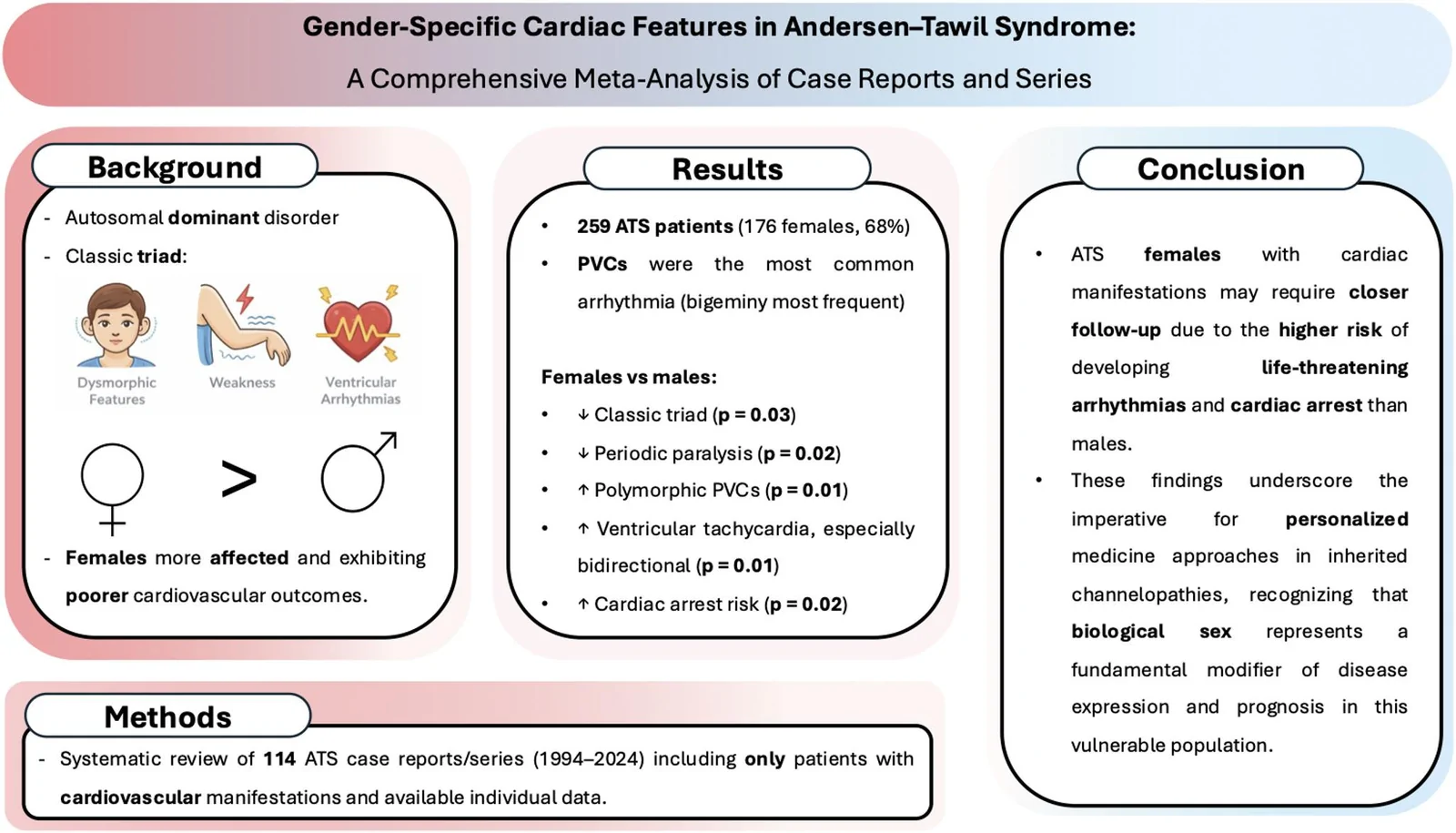
Online Abstract Figure

**Supplementary Information:**

The online version contains supplementary material available at https://doi.org/10.1007/s10840-026-02237-6.

## Introduction

Andersen-Tawil Syndrome (ATS) is a rare autosomal dominant disorder characterized by a triad of dysmorphic features, periodic muscular weakness, and frequent ventricular arrhythmias (VA) [1, 2]. The disease is most commonly caused by a loss-of-function mutation in the KCNJ2 gene, which encodes for the potassium channel Kir2.1. This channel encodes for the inward rectifier current IK1, which allows potassium ions into cardiac and skeletal myocytes, maintaining the resting membrane potential. It plays a pivotal role in the late phase of ventricular repolarization and disruptions therefore prolong the cardiac action potential, leading to the arrhythmias seen in ATS [3].

The arrhythmic phenotype of ATS includes a prolonged QT or QTU interval, a prominent U wave, premature ventricular contractions (PVCs) that manifest as couplets, bigeminy or polymorphic PVCs, and ventricular tachycardia (VT). This includes bidirectional VT (BVT) as well as Torsade de Pointes (TdP) that can degenerate to ventricular fibrillation (VF) [4]. The significant facial features include low-set ears, a broad forehead, short palpebral fissures, hypertelorism, a full nasal bridge with a bulbous tip, and maxillary and mandibular hypoplasia. In addition, the skeletal phenotypic alterations may also present dysmorphic features such as clinodactyly, syndactyly, short stature, and scoliosis [5]. Periodic paralysis can present as normokalemic, hypokalemic, or hyperkalemic. The correlation between fluctuations in potassium levels has not been linked to arrhythmic phenotypes.

Although patients with ATS often manifest a remarkable arrhythmic burden, their prognosis is generally considered benign [6]. Recently, the largest cohort of ATS patients reported that a history of unexplained syncope or documented sustained VT was associated with a higher risk of life-threatening arrhythmias (including sudden cardiac death [SCD]), aborted cardiac arrest, or hemodynamically unstable VT). In this cohort, females were the predominant group and accounted for more SCD events, although overall there was no statistically significant correlation between gender and life-threatening arrhythmias [7].

Despite these findings, important gaps remain in understanding the gender-specific arrhythmic phenotype and the clinical characteristics of patients with isolated cardiac involvement, largely due to the condition’s low prevalence estimated at just 1 case per million [8]. This meta-analysis of cases and case series describes patients with ATS who exhibit cardiac manifestations alone or combined with other disease characteristics. The aim is to provide a clearer picture of this population’s arrhythmic phenotype and clinical manifestations.

## Methodology

### Data sources & search strategy

**Fig. 1:**
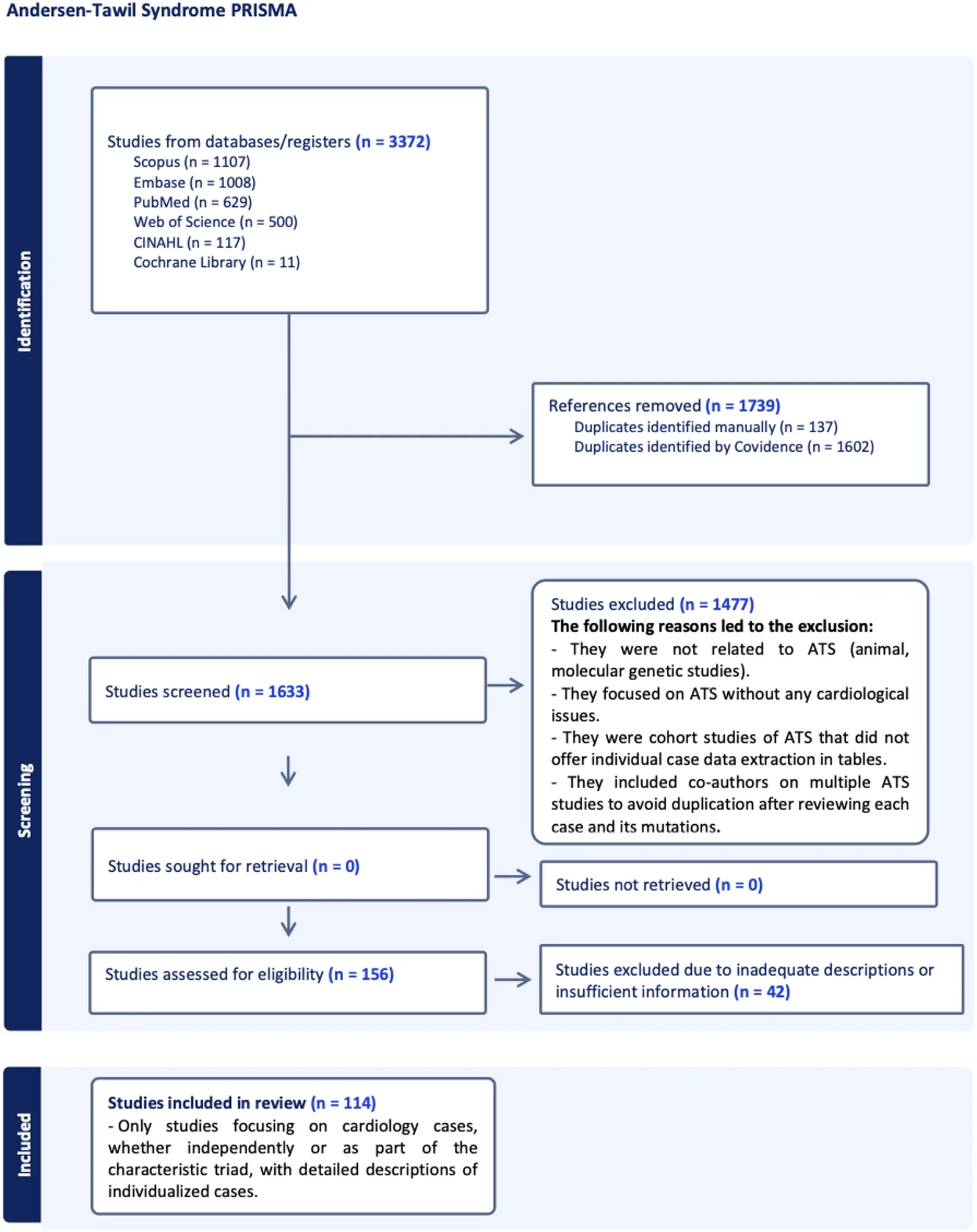
PRISMA flow chart of the screening process. The flow diagram illustrates the selection process. Initially, 3,372 studies were identified from six databases. After removing 1,739 duplicates (137 manually and 1,602 by software), 1,633 studies were screened for relevance. Of these, 1,477 were excluded for not being related to ATS cardiology cases, lacking individual case data, or containing duplicate co-authorships. A total of 156 studies were then assessed for eligibility, with 42 excluded due to inadequate descriptions or insufficient information. Finally, 114 studies focusing on cardiology cases were included

A comprehensive analysis was conducted involving 114 studies, which comprised a mix of case reports and case series (Supplemental Table 1). The electronic databases searched included PubMed, Embase (Ovid), Cochrane Library, CINAHL Complete (EBSCO), Scopus, and Web of Science, targeted from their inception to July 24, 2024. Search strategies for each database were tailored from the initial PubMed strategy, reflecting the specific indexing systems and search functionalities inherent to each platform. The search incorporated keywords associated with Andersen syndrome—including its alternative designations and genetic markers—alongside terms related to cardiac manifestations and arrhythmias. A combination of controlled vocabulary terms (like MeSH) and free-text keywords was utilized to enhance the thoroughness of the search, which focused exclusively on human studies without limitations on language or publication date. A medical librarian meticulously managed the search and data extraction. The results from all databases were aggregated and de-duplicated using the Covidence tool for further screening. Comprehensive search strategies were executed across all databases, adhering to the PRISMA-IPD statement checklist to maintain methodological rigor in the systematic literature review (Fig. 1).

### Eligibility criteria

Inclusion criteria defined that cases must exhibit Andersen syndrome with cardiac manifestations, either in isolation or in combination with dysmorphic features (DF) and/or periodic paralysis (PP).

A thorough review of each article and its authors was conducted to prevent patient duplication. Cohort studies or case series lacking detailed patient characteristics or sharing co-authors in multiple cohorts were excluded, prioritizing data collection on individual patient characteristics. Illustrations of the reported electrocardiograms (ECGs) were utilized to fill in information gaps, especially in cases where interpretation was not described. The ECG analysis was conducted using an EP Caliper software (EP Calipers 3 software; Version 2.5.2, EP Studios Software), and interpretations were provided by two electrophysiologists who evaluated arrhythmic features, as well as QT and QT-U interval measurements. The QTc interval was considered prolonged if it exceeded ≥ 480 milliseconds in accordance with guidelines for the diagnosis and management of inherited primary arrhythmia syndromes [9]. High-burden polymorphic ventricular contractions (PVCs) were described based on 24-hour Holter monitoring results when they showed more than ≥ 15% PVCs, a total of over 15,000 PVCs, or if the authors specifically classified them as high-burden [1–3]. Although there is no universally accepted definition of a high PVC burden, the 2022 ESC Guidelines consider a threshold of ≥ 10% clinically relevant for PVC-induced cardiomyopathy [10]. Observational studies and expert reviews, including Marcus et al. [11], generally cite 10–15% as the range of concern. Baman et al. [12] reported a strong association between cardiomyopathy and higher PVC burdens (≥ 24%), though cases were also seen at levels as low as 10–15%. Similarly, Dukes et al. [13] found that burdens exceeding 10% were linked to an increased risk of heart failure and mortality. In patients with Andersen–Tawil syndrome, PVC burden often surpasses these thresholds, yet no specific cutoff has been established. We therefore applied this guideline- and evidence-based value to define high burden, while acknowledging the variability in definitions.

Patients were selected by three methods. First if they underwent genetic analysis and had a positive mutation in the KCNJ2 gene documented. Second, if they had either had a clinical diagnosis with a negative genetic analysis but who met the clinical diagnostic criteria for ATS. Third, due to the rarity of ATS, case reports were included even if they lacked genetic analysis, as long as the publication established the clinical diagnostic criteria for ATS (*n* = 11).

All KCNJ2 variants were annotated using HGVS nomenclature (NM_000891.3) and systematically cross-referenced with ClinVar [14], HGMD [15], OMIM [16], and LOVD [17]. When available, functional evidence from patch-clamp electrophysiology or trafficking assays was incorporated. Final classification followed ACMG/AMP 2015 guidelines [18], integrating evidence from population frequency, segregation analysis, database submissions, and experimental data. Variants were categorized as pathogenic, likely pathogenic, variants of uncertain significance (VUS), or benign/likely benign original reports often lacked uniform classification or used non-standard criteria. This process occasionally differed from the author’s original designation or from isolated database entries, particularly in rare substitutions with limited segregation data. Thus, the final counts of pathogenic, likely pathogenic, and VUS variants presented in this analysis represent a harmonized consensus reclassification, not a direct collation of author-reported categories.

For cases without molecular testing, diagnosis relied on established clinical features and were designated as “clinical ATS.” Individuals who underwent sequencing but tested negative for KCNJ2 were classified as “KCNJ2 negative”, provided their clinical phenotype fulfilled accepted diagnostic criteria. In a small subset, mutation positivity was inferred rather than directly confirmed: in one family, the grandmother and mother of a proband were considered obligate carriers of the pathogenic R218W variant based on pedigree analysis, as the proband and both of her daughters had confirmed mutations and overlapping phenotypic expression. In another family, the mother of a proband carrying the A304D variant, classified as likely pathogenic, was assumed to have harbored the same variant; her sudden cardiac death was attributed to ATS on the basis of segregation with the proband’s confirmed mutation and the presence of a compatible arrhythmic phenotype. These inferences were necessary due to the absence of direct but were applied conservatively and transparently.

All other analyses, including genotype–phenotype correlations, clinical outcomes, and mortality events, were performed strictly on cases with documented genetic data or clinical diagnoses explicitly reported by the primary authors. Large structural alterations, including complete KCNJ2 deletions, were considered pathogenic across ACMG/AMP-based reclassification and major databases; However, to avoid conflation with single-nucleotide and small indel variants, they were reported separately.

### Statistical analysis

Statistical analysis was executed using SPSS version 25 (IBM, Italy). Cases were stratified by gender to facilitate comparisons of their cardiac and morphological profiles and manifestations of periodic paralysis. Descriptive statistics were generated using the reported data for each variable. Normally distributed continuous variables were expressed as means with standard deviations (SD), while both normally and non-normally distributed variables were compared using Student’s t-test and the Mann–Whitney U-test, respectively. Categorical variables were evaluated using the Chi-squared test or Fisher’s exact test.

## Results

### Study population

**Fig. 2:**
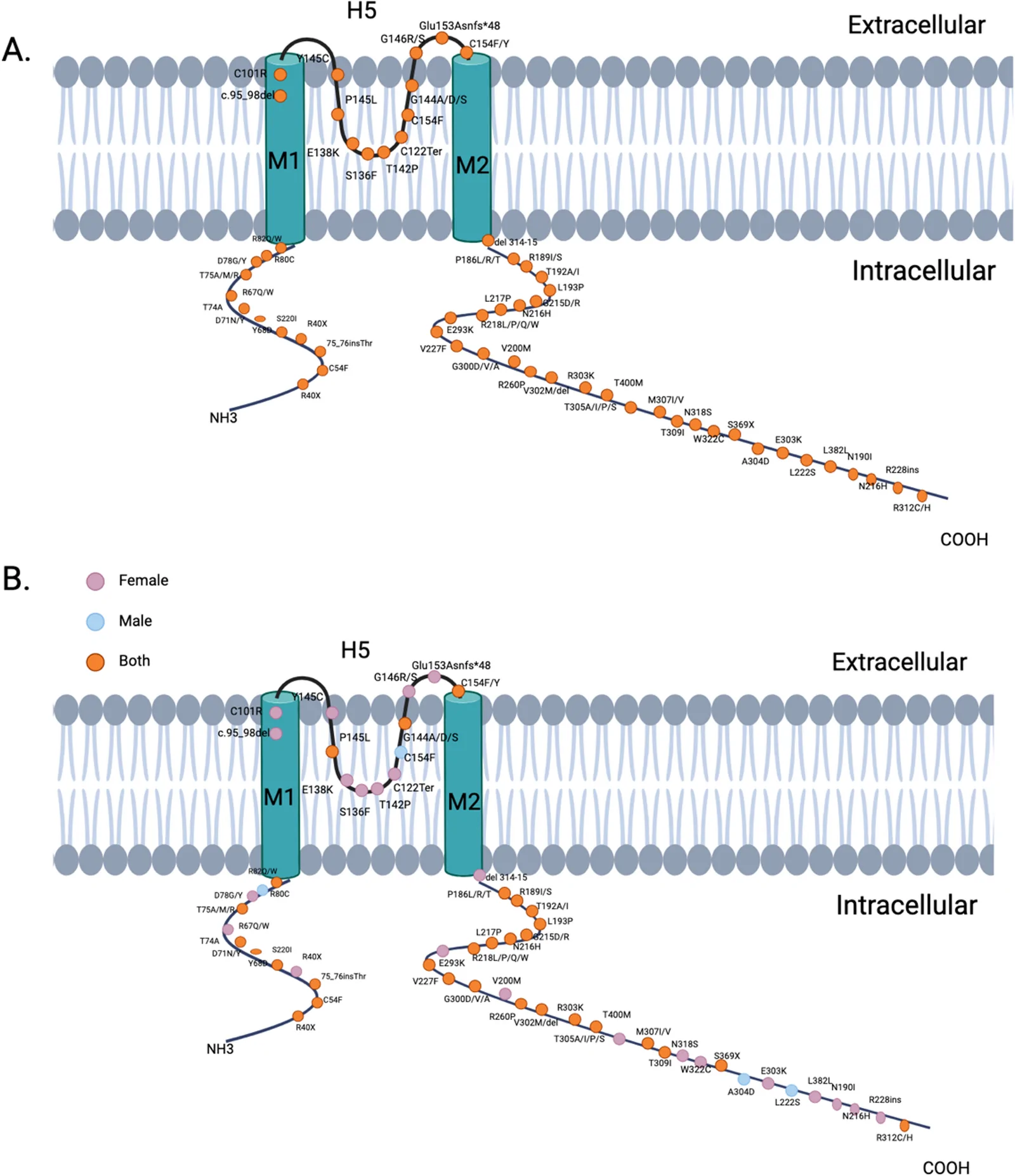
Distribution of genetic variants along the KCNJ2 gene and sex-specific occurrence. (**A**) Schematic representation of the KCNJ2 gene, showing the relative positions of known genetic variants along the amino acid sequence. Orange circles indicate the locations of amino acid changes or deletions. (**B**) The same schematic with sex-specific occurrence: pink represents variants observed exclusively in females, blue for males, and orange for variants found in both sexes. NH3: amino terminus; COOH: carboxyl terminus; H5: helix 5; M1, M2: transmembrane domains

The population included 259 patients, of whom 176 are female (68%) and 199 were reported as probands. Among 259 ATS patients, 244 (94%) harbored a *KCNJ2* mutation (Fig. 2). Of the genetically positive cases, 106 (41%) variants were classified as pathogenic, 90 (35%) as likely pathogenic, 46 (18%) as variants of uncertain significance (VUS). Recurrent substitutions such as R218W, R312C, M307I, and G146R were consistently annotated as pathogenic across databases, a classification further supported by functional assays demonstrating loss of Kir2.1 current density or impaired trafficking. By contrast, rare substitutions such as T74A, L22S, and C122Y remained VUS due to absent segregation data and conflicting in silico predictions. The remaining patients comprised 3 individuals with negative *KCNJ2* sequencing despite fulfilling ATS diagnostic criteria, 11 diagnosed clinically without molecular testing. Importantly, 2 cases evidenced a complete *KCNJ2* deletion. While these deletions were considered pathogenic for ATS, they were not included in the pathologic single-variant tally to preserve separation between sequence-level and structural variants (Supplemental Table 2).

### Dysmorphic features and periodic paralysis

Morphologic features of ATS were reported in 253 cases. The classic triad was the most frequent presentation (*n* = 128), followed by the arrhythmia with dysmorphic features (*n* = 66) and arrhythmia with periodic paralysis (*n* = 28). An additional 32 patients presented with isolated cardiac manifestations, all of whom had genetic confirmation: pathogenic (*n* = 15), likely pathogenic (*n* = 6) or VUS (*n* = 9) variants, one unspecified KCNJ2 variant, and one KCNJ2 gene deletion. Female patients were less likely than males to present with the classic triad (46% vs. 61%, respectively [*p* = 0.03; OR = 0.54; CI = 0.32–0.93]).

Of the 253 analyzed cases, 192 reported dysmorphic anomalies compatible with ATS. These anomalies were more frequently present in males than females (80% vs. 73%). However, among affected individuals, females tended to display a greater number of distinct dysmorphic features (mean 4 ± 2) compared with males (mean 3 ± 2). The three most common features include micrognathia with 120 cases, clinodactyly in 72 patients, and low-set ears in a total of 70 cases. Less common features include cleft lip and palate (*n* = 8), followed by hand abnormalities, single palmar crease (*n* = 4), and camptodactyly (*n* = 2).

Although no statistically significant sex differences were identified (all 95% confidence intervals crossed 1; all *P* > 0.05), several non-significant sex-related trends were observed. Females showed higher proportions of microcephaly, hypertelorism, short palpebral features, broad nasal bridge, bulbous tip nose, low-set ears, micrognathia, cleft lip and palate, scoliosis, syndactyly, and brachydactyly. In contrast, males tended to have higher frequencies of short stature, broad forehead, dental abnormalities, thin upper lip, and single palmar crease. Clinodactyly and camptodactyly occurred at identical frequencies in both sexes (Table 1).

**Table 1:** Characteristics dysmorphic features of the study population

	Evaluated cases^†^ (*n*) (*n* = 253)	Affected cases (*n*)	Females, *n* (%) (*n* = 172)	Males, *n* (%) (*n* = 81)	OR	95% CI	*P* value ‡
**Microcephaly**	217	16	14 (8)	2 (2)	3.27	0.72–14.8	0.10
**Short stature**	217	63	42 (24)	21 (26)	0.82	0.44–1.54	0.55
**Broad forehead**	217	23	14 (8)	9 (11)	0.64	0.26–1.58	0.33
**Hypertelorism**	205	58	41 (24)	17 (21)	1.03	0.52–2.00.52.00	0.93
**Short palpebral features**	217	11	9 (5)	2 (2)	2.02	0.42–9.65	0.36
**Broad nasal bridge**	205	26	22 (13)	4 (5)	2.57	0.84–7.80	0.08
**Bulbous tip nose**	206	13	12 (7)	1 (1)	5.41	0.68–42.5	0.07
**Low set ears**	205	70	49 (28)	21 (26)	0.98	0.52–1.84	0.95
**Micrognathia**	217	120	83 (48)	37 (46)	0.95	0.53–1.71	0.88
**Dental abnormalities**	218	32	20 (12)	12 (15)	0.68	0.31–1.49	0.33
**Thin upper lip**	217	17	11 (6)	6 (7)	0.78	0.27–2.22	0.64
**Cleft lip and palate**	218	8	6 (3)	2 (2)	1.32	0.26–6.73	0.73
**Scoliosis**	217	13	10 (6)	3 (4)	1.48	0.39–5.59	0.55
**Single palmar crease**	217	4	2 (1)	2 (2)	0.43	0.05–3.11	0.39
**Clinodactyly**	217	72	49 (28)	23 (28)	0.89	0.48–1.65	0.73
**Syndactyly**	217	25	21 (12)	4 (5)	2.50	0.82–7.60	0.09
**Brachydactyly**	217	11	9 (5)	2 (2)	2.02	0.42–9.65	0.36
**Camptodactyly**	217	2	1 (1)	1 (1)	0.43	0.27–7.03	0.54

^**†**^Values reported are using the available cases (n) that described the morphologic anomaly from the overall population.‡ P values represented the comparison of females vs. males for each feature

Information regarding periodic paralysis was reported in 254 cases of the 259 included cases. Periodic paralysis affected a total of 156 patients, of whom males had 1.93 times higher odds of present periodic paralysis than females (*p* = 0.02). In terms of the age of periodic paralysis onset, both had a similar age of first episode presentation (10 ± 9 in females vs. 10 ± 8 in males).

### Cardiovascular manifestations

259 reported at least one cardiovascular symptom compatible with ATS. The average age of onset was similar in females and males (17 ± 12 vs. 17 ± 14, respectively). Syncope was reported in 71 patients and was more common in females than males, with nearly double the odds (32% vs. 18% [*p* = 0.03; OR = 1.99; CI = 1.04–3.82]).

Of 202 cases that reported information regarding the QTU interval or a prominent U wave, 140 demonstrated this feature. 221 patients provided information about prolonged QTc intervals, either by providing an ECG for measurement or through the authors noting that QTc was prolonged. Among these, 98 cases (44%) had a prolonged QTc interval, with 68% of those being females. In a secondary analysis we included only patients for whom actual QTc values were available, yielding data for 177 of the 221 patients. The results indicated that females had a longer QTc measurement (451 ± 52 ms) compared to males (430 ± 57 ms) (*p* = 0.008). However, when considering the definition of a prolonged QTc interval ≥ 480 ms, only 48 patients met this criterion, of which 36 were females (75%).

The entire study population reported at least one type of VA, with PVCs being the most commonly reported anomaly. Of the 255 cases that reported PVCs, females and males had similar percentages (Both with 88%). Females reported a higher PVC burden (24%) than males (14%). Bigeminy was the most common type of PVC described with 128 cases, both genders with a similar percentage of affected cases (Table 2). Couplets were described in 83 patients, being more common in females than males (36% vs. 24%, respectively [*p* = 0.02; OR = 2.04; CI = 1.09–3.80]). A total of 74 patients reported polymorphic PVCs, being present more in females (33%) than males (19%) (*p* = 0.01, OR = 2.24; CI = 1.17–4.27).

**Table 2:** Cardiac characteristics of the study population

	Evaluated cases^†^ (*n*) (*n* = 259)	Affected cases (*n*)	Affected Females, *n* (%) (*n* = 176)	Affected Males, *n* (%) (*n* = 83)	OR	95% CI	*P* value‡
**Syncope** ^♦^	246	71	56 (32)	15 (18)	1.99	1.04–3.82	**0.03**
**Family history of SCD**	259	39	29 (16)	10 (12)	1.44	0.66–3.11	0.35
**QTU Positive**	202	140	99 (56)	41 (65)	1.32	0.70–2.50	0.38
**QTc ≥ 480 ms**	177	48	67 (38)	31 (49)	1.50	0.70–3.01	0.28
**PVC**	255	228	155 (88)	73 (88)	1.06	0.45–2.47	0.89
**High burden PVC**	250	54	42 (24)	12 (14)	1.90	0.93–3.85	0.07
**Bigeminy**	207	128	86 (49)	42 (51)	1.00	0.55–1.82	0.98
**Couplets**	205	83	63 (36)	20 (24)	2.04	1.09–3.80	**0.02**
**Polymorphic PVC**	223	74	58 (33)	16 (19)	2.24	1.17–4.27	**0.01**
**VT**	256	184	131 (74)	53 (64)	1.66	0.94–2.94	0.07
**nsVT**	239	137	99 (56)	38 (46)	1.48	0.85–2.57	0.15
**sVT**	240	38	28 (16)	10 (12)	1.32	0.60–2.89	0.47
**BVT**	238	111	85 (48)	26 (31)	2.05	1.16–3.61	**0.01**
**Polymorphic VT**	237	56	41 (23)	15 (18)	1.28	0.66–2.51	0.45
**TdP**	239	12	8 (5)	4 (5)	0.91	0.26–3.12	0.88
**VF**	246	36	28 (16)	8 (9)	1.75	0.78–4.03	0.18
**NFCA**	259	25	22 (13)	3 (3)	3.81	1.10–13.1	**0.02**
**SCD**	259	12	9 (5)	3 (3)	1.43	0.37–5.45	0.59
**ICD**	259	50	38 (22)	12 (14)	1.62	0.80–3.31	0.17

^**†**^Values are (n) using the available cases that described each anomaly from the overall population (*n* = 259).‡ P values represented the comparison of females vs. males for each feature.^♦^ Syncope reported as binary; mechanism not specified; vasovagal component cannot be excluded.BVT = Bidirectional Ventricular Tachycardia; ICD = Implantable Cardioverter Defibrillator; NFCA = Non-fatal Cardiac Arrest; PVCs = Premature Ventricular Contractions; SCD = Sudden Cardiac Death; TdP = Torsade de Pointes; VF = Ventricular Fibrillation; VT = Ventricular Tachycardia; nsVT = Non-sustained Ventricular Tachycardia; sVT = Sustained Ventricular Tachycardia

VT was presented in 184 cases; this was seen predominantly in females than males (74% vs. 64% respectively). 137 of the patients reported non-sustained VT episodes, more commonly presented in females (56%) than males (46%). Only 38 evidenced sustained VT, of which 28 were females and 10 males. BVT was the most reported type of VT, with 111 cases, more frequently seen in females than males (48% vs. 31%, respectively [*p* = 0.01; OR = 2.05; CI = 1.16–3.61]). Polymorphic VT was present in 56 cases with almost similar percentages in both groups (23% in females vs. 18% in males). TdP was reported in 12 cases with a similar affected percentage in both groups (Table 2). VF was reported more in females (16%) than males (9%).

Family history of SCD was infrequently reported, with only 39 patients or families disclosing such a history. Across all included cases, 37 individuals experienced a cardiac arrest, whether fatal or non-fatal. A higher proportion of these events occurred in females compared with males (31 vs. 6, respectively; *p* = 0.02; OR = 2.74; 95% CI: 1.09–6.86). Nonfatal cardiac arrest (NFCA) was reported in 25 cases, again predominantly in females (*n* = 22 vs. *n* = 3; *p* = 0.02; OR = 3.8; 95% CI: 1.10–13.1). The average age of NFCA in females (25 ± 14 years) and males (27 ± 15 years) was similar. Rates of SCD did not reach statistical significance, though 9 of 12 reported cases were female (average age 34 ± 12 years in females vs. 35 ± 11 years in males). The majority of arrest events occurred in patients harboring pathogenic or likely pathogenic KCNJ2 variants. However, a subset of events arose outside this group. Mutation positivity was assumed in three relatives: two possible carriers (the mother and grandmother of a proband) with a confirmed R218W mutation, corroborated by pedigree segregation, and the mother of a proband with A304D, whose sudden cardiac death was attributed to ATS on the basis of family history and arrhythmic phenotype. Two NFCA cases tested negative for KCNJ2 but fulfilled diagnostic clinical criteria, and two additional NFCA cases were diagnosed clinically without molecular testing. Finally, one reported SCD case was diagnosed on clinical grounds in the absence of genetic data. These cases highlight the heterogeneity of ascertainment and the need for cautious interpretation of mortality attribution in the absence of confirmed genotyping.

A total of 50 patients had an ICD implanted (*n* = 38 females vs. *n* = 12 males). ICD was placed for primary prevention in 34 cases, 25 of them were females. The rest were implanted as secondary prevention (*n* = 16) following an aborted cardiac arrest, of which 13 were placed in females. 13 patients reported appropriate ICD discharges, and one patient reported inappropriate discharges. Among those who had an implanted ICD, 68% reported a history of syncope (p = < 0.001). Patients who underwent ICD implantation were also more likely to report an episode of sustained VT compared to those who did not undergo ICD implantation (35% vs. 11%, respectively [p = < 0.001; OR = 4.36; CI = 2.12–9.40]). Among the total 50 ICD recipients, 14 carried pathogenic KCNJ2 variant, 19 had a likely pathogenic variant, 6 were classified as VUS. The rest of patients included 5 patients with KCNJ2-positive without specification of the underlying variant, 5 diagnosed clinically without genetic testing, and 1 patient with negative KCNJ2 result but meeting clinical diagnostic criteria for ATS.

## Discussion

**Fig. 3:**
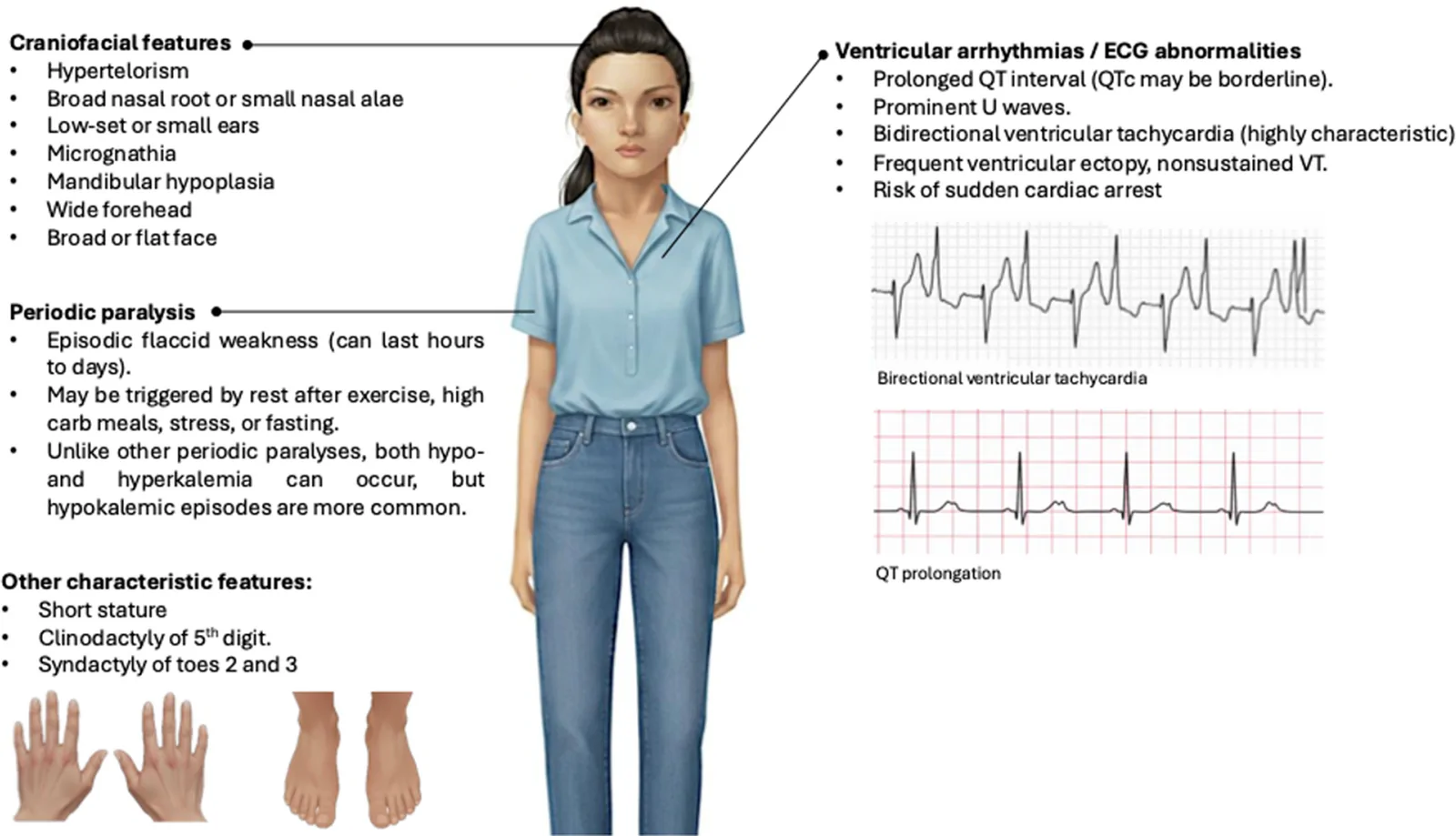
Schematic representation of Andersen–Tawil syndrome (ATS) highlighting its classic clinical features. The diagram illustrates characteristic craniofacial features, periodic paralysis, common ventricular arrhythmias and ECG abnormalities are also shown, as well as distinctive skeletal features

Andersen-Tawil syndrome is a rare, autosomal-dominant disease with an unknown frequency known to cause VA. It is distinguished by extracardiac manifestations, including periodic paralysis and a mixture of skeletal and facial dysmorphisms [1, 2] (Fig. 3).

The disease is caused by a loss of function in the KCNJ2 gene, which encodes for the inward rectifier potassium channel Kir2.1, responsible for the IK1 potassium current [3]. This comprehensive meta-analysis of ATS patients with cardiac involvement reveals profound gender disparities that illuminate fundamental differences in how inherited QT-prolonging arrhythmias manifest across biological sexes. These findings reveal that ATS occupies a unique and concerning position within the spectrum of inherited QT-prolonging arrhythmias, demonstrating the most pronounced female-predominant arrhythmic risk profile among channelopathies.

ATS is still classified as long QT-syndrome (LQTS) type 7, despite multiple objections from experts given of the lack of consistent QT/QTc prolongation [19]. Our ATS cohort demonstrates a distinct gender-specific arrhythmic pattern that both aligns with and diverges from established long QT syndrome (LQTS) paradigms. While classic LQTS shows that females constitute approximately 56–70% of affected patients with higher disease penetrance than males [20], our ATS data revealed QTc prolongation in 44% of cases. When applying the cutoff of ≥ 480 ms, only 19% of patients had a prolonged QTc, with a pronounced female predominance (75%). This subgroup exhibited a substantially elevated arrhythmic risk, underscoring the need for exceptional caution with QT-prolonging medications [21].

The unique gender risk profile in ATS, where females exhibit significantly longer QTc intervals (451 ± 52ms vs. 430 ± 57ms), likely reflects estrogen’s multifaceted effects on cardiac ion channels. Estrogen decreases potassium channel current, particularly affecting the rapidly activating delayed rectifier current (IKr), and may enhance L-type calcium current density [22, 23]. In ATS patients with already compromised inward rectifier current (IK1) due to KCNJ2 mutations, these estrogen-mediated effects create a “perfect storm” for arrhythmogenesis by further compromising repolarization reserve.

Recent computational modeling studies demonstrate that females exhibit consistently longer action potentials and greater susceptibility to early afterdepolarizations compared to males, with IKr loss-of-function leading to early afterdepolarizations in 65.8% of female models versus only 22.8% of male models [24]. These findings provide mechanistic support for the clinical observations in our ATS cohort, where females demonstrated higher rates of polymorphic VA (23% vs. 18%), and increased BVT (48% vs. 31%) than males, likely reflecting estrogen’s pronounced effects on already compromised KCNJ2-mediated repolarization reserve through inhibition of multiple potassium currents and enhancement of calcium handling. The higher prevalence of BVT in ATS females carries particular significance, as this arrhythmia pattern is pathognomonic of ATS and associated with a high risk of degeneration to VF [25].

The hormonal fluctuations throughout the female reproductive cycle may contribute to the increased arrhythmic vulnerability observed in ATS females. Studies demonstrate that QT intervals vary with menstrual cycle phases, with shorter durations during the luteal phase attributed to increased progesterone levels [26, 27]. This sex-specific vulnerability appears to intensify across the female reproductive lifespan, with menstrual cycle variations affecting QT intervals and pregnancy representing a particularly high-risk period for women with inherited arrhythmia syndromes [26]. While no inherited arrhythmia syndrome represents an absolute contraindication to pregnancy, the hormonal changes and hemodynamic stress of pregnancy may increase arrhythmic risk [28]. For ATS females, who already demonstrate heightened vulnerability to life-threatening arrhythmias, pregnancy requires intensive monitoring and potentially modified therapeutic approaches. These findings position ATS as one of the more sexually dimorphic inherited channelopathy and necessitate fundamental revisions to risk stratification algorithms, with female sex emerging as an independent risk factor requiring sex-specific therapeutic approaches and intensified surveillance strategies.

The relative protection observed in ATS males likely stems from testosterone’s opposing effects on cardiac repolarization. Testosterone increases outward potassium currents (IKr, IKs, and IK1), potentially providing partial compensation for KCNJ2 loss-of-function mutations [26, 29]. This hormonal protection may explain why males in our cohort demonstrated shorter QTc intervals and lower rates of life-threatening arrhythmias despite carrying the same genetic mutations.

The protective effect of testosterone becomes particularly evident when examining age-related patterns across inherited channelopathies. In LQTS, males show higher event rates before puberty, but females experience increased risk after adolescence, coinciding with hormonal maturation [20, 30]. Our ATS data shows similar ages of cardiac symptom onset between sexes (17 ± 12 vs. 17 ± 14 years), supporting the well-established pattern in inherited channelopathies where gender differences emerge primarily after puberty. This finding reinforces existing knowledge that pubertal hormonal changes serve as critical triggers for phenotype expression in channelopathies, with females experiencing heightened vulnerability to arrhythmic events during reproductive years due to estrogen’s effects on ion channel function.

Traditional risk factors such as syncope history and sustained VT remain important predictors in ATS, consistent with the Mazzanti et al. study, which identified syncope (HR: 4.54; *p* = 0.02) and sustained VT (HR: 9.34; *p* = 0.001) as significant predictors of life-threatening arrhythmic events [7]. Although syncope was frequently reported in our cohort (32% of females vs. 18% of males; *p* = 0.03), most reports did not specify the underlying mechanism, and a vasovagal component cannot be excluded. Nonetheless, all included patients had documented cardiac abnormalities, and syncope was considered clinically relevant to the arrhythmic phenotype. Importantly, studies have demonstrated that despite frequent ventricular ectopy and non-sustained VT in up to 88% of KCNJ2 mutation carriers, the incidence of SCD remains relatively low compared to other inherited arrhythmia syndromes [31]. Our analysis reveals that female sex is associated with a 2.74-fold increased risk of cardiac arrest (*p* = 0.02). This observation points to a possible sex-related vulnerability in ATS, though it should be interpreted with caution. In the absence of systematic family cascade screening and complete genetic confirmation for all cases, precise estimates of penetrance and variant-specific risk remain uncertain. Nonetheless, we believe it is important to report this signal, as it is consistent with the documented female predominance in ATS cohorts (61–68%) and with the characteristically prolonged QTc intervals observed in females (451 ± 52 ms), which may predispose to polymorphic arrhythmias [25]. Rather than serving as definitive risk estimates or immediate clinical recommendations, our findings highlight the need for careful consideration of sex in future prospective studies and in genetically confirmed, family-based cohorts to clarify its role in risk stratification.

Beta-blocker therapy, while beneficial for reducing arrhythmic burden, requires careful titration in ATS females given their baseline slower heart rates and longer repolarization times [7, 9, 32]. The combination of beta-blockers with flecainide has shown promise in reducing arrhythmia burden in ATS patients [33], though optimal therapeutic approaches may require sex-specific considerations given the higher baseline arrhythmic risk in females.

Our analysis reveals a significant gender disparity in implantable cardioverter-defibrillator (ICD) utilization among ATS patients, with females receiving proportionally more implantations (38 vs. 14 devices) and constituting 74% (25/34) of primary prevention recipients. This pattern directly reflects the elevated arrhythmic burden and clinical event rates observed in female ATS patients, including their higher cardiac arrest risk. The strong association between sustained VT history and ICD implantation (OR = 4.32, *p* < 0.001) supports current risk stratification approaches [7], yet the predominance of female recipients suggests that sex may also influence management patterns in ATS. This stands in contrast to broader cardiology practice, where women are typically underrepresented in device implantation despite comparable indications [34]. While these findings should be interpreted cautiously in light of incomplete genetic data, they highlight the need for further study before sex-specific recommendations regarding ICD allocation can be made. In this context, and given the historically higher complication rates associated with transvenous ICDs in women [35], the availability of subcutaneous ICDs (S-ICDs) shown to provide equivalent safety and efficacy across sexes [36]. This positions S-ICDs as a strategically preferable option for female ATS patients, particularly given their documented vulnerability to polymorphic arrhythmias and prolonged repolarization [7].

## Implications for future research

Our findings highlight critical knowledge gaps requiring targeted investigation. The interaction between specific KCNJ2 mutations and sex hormones warrants detailed study, as certain variants may exhibit sex-dependent penetrance and expressivity. Prospective studies should systematically evaluate hormonal influences on ATS phenotype, including assessment of arrhythmic burden during different menstrual cycle phases and pregnancy-related complications.

The development of sex-specific risk prediction models for ATS represents an urgent clinical need to prevent unnecessary procedural and long-term complications. Traditional risk stratification tools developed primarily in male-predominant populations may inadequately capture the unique risk profile of ATS females, necessitating the development of gender-tailored algorithms that incorporate hormonal factors, menstrual status, and reproductive history.

## Study limitations

This meta-analysis has several limitations, primarily reflecting the heterogeneity and incompleteness of the published source data. Phenotypic characterization was variably reported, with inconsistent documentation of dysmorphic features, neuromuscular involvement, and detailed arrhythmic findings such as electrophysiologic parameters, structural abnormalities, and arrhythmia subtypes. Larger cohorts describing outcomes like cardiac arrest and SCD could not be included because disaggregated case-level data were unavailable, limiting the precision of outcome estimates.

Uncertainty also arises from the classification of mutation status in selected cases. In one family reported by Tully et al. [37], mutation positivity was inferred in the proband’s mother and grandmother based on segregation with the pathogenic R218W variant, although neither relative underwent direct testing. In Borowska et al. [38], the proband carried the A304D variant, classified as likely pathogenic, while his mother who died suddenly at age 28 was assumed to share the variant on the basis of family history, segregation, and arrhythmic phenotype, though without molecular confirmation. Two additional NFCA cases were clinically diagnosed as ATS despite negative KCNJ2 sequencing [39, 40] and two more were diagnosed solely on clinical grounds without genetic testing [41, 42]. Finally, one case of SCD reported by Peters et al. [43] was attributed to ATS despite negative KCNJ2 sequencing, based on the diagnostic clinical presentation.

These inclusions were necessary to capture the full clinical spectrum of ATS, but they inevitably introduce the possibility of misclassification bias, particularly with respect to mortality outcomes. As such, the reported estimates should be regarded as exploratory signals rather than definitive risk measures. They highlight the urgent need for larger multicenter studies with systematic genotyping, segregation analysis, and longitudinal follow-up to clarify the prognostic significance of specific variants and refine risk stratification in ATS.

## Conclusion

This meta-analysis provides compelling evidence for the most pronounced gender disparity reported among inherited QT-prolonging arrhythmias, with ATS females demonstrating exceptional vulnerability to life-threatening events. The observed differences in QTc prolongation, VA complexity, and cardiac arrest risk necessitate fundamental revisions to ATS management paradigms, with female sex emerging as a critical determinant of outcomes requiring sex-specific risk stratification and therapeutic approaches. These findings underscore the imperative for personalized medicine approaches in inherited channelopathies, recognizing that biological sex represents a fundamental modifier of disease expression and prognosis in this vulnerable population.

## Supplementary Information

Below is the link to the electronic supplementary material.

Supplementary File 1 (DOCX 55.1 KB)

Supplementary File 2 (DOCX 4.01 MB)

## Data Availability

Data is provided within the supplementary information files.

## Abbreviations

ATS: Andersen-Tawil Syndrome
DF: Dysmorphic features
IK1: Inward rectifier current
LQTS: Long QT syndrome
PP: Periodic paralysis
PVCs: Premature ventricular contractions
SCD: Sudden cardiac death
TdP: Torsade de Pointes
VA: Ventricular arrhythmias
VT: Ventricular Tachycardia
VF: Ventricular fibrillation
BVT: Bidirectional Ventricular Tachycardia
