# Supplementary material for: Gender-specific cardiac features in Andersen–Tawil syndrome: a comprehensive meta-analysis of case reports and series
Source: J Interv Card Electrophysiol. 2026 Jan 14;69(6):1241–52. doi: 10.1007/s10840-026-02237-6 (PMC13645902; doi:10.1007/s10840-026-02237-6)
Supplement: Supplementary file 1 [file 10840_2026_2237_MOESM1_ESM.docx]

Supplementary Table 2 – Reported KCNJ2 Variants in Andersen–Tawil Syndrome (n=259)

| Patient number | Mutation | Molecular Consequence | ClinVar Classification | HGMD Classification | Other Databases | ACMG / Interpretation |
| --- | --- | --- | --- | --- | --- | --- |
| 1. | KCNJ2 Pos | — | Pathogenic (variant not specified) | DM/Reported | Reported positive | Pathogenic (reported) |
| 2. | R218W | Missense | Pathogenic | DM | LOVD/ClinGen | Pathogenic |
| 3. | R218W | Missense | Pathogenic | DM | LOVD/ClinGen | Pathogenic |
| 4. | — | — | NA | NA | Clinical ATS (no variant) | Clinical ATS diagnosis |
| 5. | — | — | NA | NA | Clinical ATS (no variant) | Clinical ATS diagnosis |
| 6. | R218W | Missense | Pathogenic | DM | LOVD/ClinGen | Pathogenic |
| 7. | p.Ala91_Leu94del | In-frame deletion | Likely Pathogenic | DM | LOVD | Likely Pathogenic |
| 8. | p.Ala91_Leu94del | In-frame deletion | Likely Pathogenic | DM | LOVD | Likely Pathogenic |
| 9. | p.Ala91_Leu94del | In-frame deletion | Likely Pathogenic | DM | LOVD | Likely Pathogenic |
| 10. | KCNJ2 Neg | — | NA | NA | Clinical ATS (no variant) | Clinical ATS diagnosis |
| 11. | R67W | Missense | VUS / Likely Pathogenic | DM | LOVD | Likely Pathogenic |
| 12. | R218W | Missense | Pathogenic | DM | LOVD/ClinGen | Pathogenic |
| 13. | S136F | Missense | Pathogenic | DM | LOVD | Pathogenic |
| 14. | R40X | Nonsense | Pathogenic | DM | LOVD | Pathogenic |
| 15. | R67Q | Missense | VUS | DM | LOVD | Likely Pathogenic |
| 16. | G146S | Missense (pore-loop) | Pathogenic | DM | LOVD | Pathogenic |
| 17. | T192A | Missense | VUS | DM | LOVD | Likely Pathogenic |
| 18. | T192A | Missense | VUS | DM | LOVD | Likely Pathogenic |
| 19. | R218Q | Missense (hotspot) | Pathogenic | DM | LOVD/ClinGen | Pathogenic |
| 20. | R218Q | Missense (hotspot) | Pathogenic | DM | LOVD/ClinGen | Pathogenic |
| 21. | R218Q | Missense (hotspot) | Pathogenic | DM | LOVD/ClinGen | Pathogenic |
| 22. | R218Q | Missense (hotspot) | Pathogenic | DM | LOVD/ClinGen | Pathogenic |
| 23. | R218W | Missense | Pathogenic | DM | LOVD/ClinGen | Pathogenic |
| 24. | R218W | Missense | Pathogenic | DM | LOVD/ClinGen | Pathogenic |
| 25. | R218W | Missense | Pathogenic | DM | LOVD/ClinGen | Pathogenic |
| 26. | R218W | Missense | Pathogenic | DM | LOVD/ClinGen | Pathogenic |
| 27. | R218W | Missense | Pathogenic | DM | LOVD/ClinGen | Pathogenic |
| 28. | R218W | Missense | Pathogenic | DM | LOVD/ClinGen | Pathogenic |
| 29. | R218W | Missense | Pathogenic | DM | LOVD/ClinGen | Pathogenic |
| 30. | R80C | Missense | Pathogenic | DM | LOVD | Pathogenic |
| 31. | R82Q | Missense | Pathogenic | DM | LOVD | Pathogenic |
| 32. | R82W | Missense | Pathogenic | DM | LOVD | Pathogenic |
| 33. | R218W | Missense | Pathogenic | DM | LOVD/ClinGen | Pathogenic |
| 34. | R218W | Missense | Pathogenic | DM | LOVD/ClinGen | Pathogenic |
| 35. | R228ins | In-frame insertion | Likely Pathogenic | DM | LOVD | Likely Pathogenic |
| 36. | T309I | Missense | VUS | DM | LOVD | Likely Pathogenic |
| 37. | R67W | Missense | VUS / Likely Pathogenic | DM | LOVD | Likely Pathogenic |
| 38. | R67W | Missense | VUS / Likely Pathogenic | DM | LOVD | Likely Pathogenic |
| 39. | R67W | Missense | VUS / Likely Pathogenic | DM | LOVD | Likely Pathogenic |
| 40. | R67W | Missense | VUS / Likely Pathogenic | DM | LOVD | Likely Pathogenic |
| 41. | G300V | Missense | Likely Pathogenic | DM | LOVD | Likely Pathogenic |
| 42. | G300V | Missense | Likely Pathogenic | DM | LOVD | Likely Pathogenic |
| 43. | 95_98del | In-frame deletion | Pathogenic | DM | LOVD | Pathogenic |
| 44. | S136F | Missense | Pathogenic | DM | LOVD | Pathogenic |
| 45. | G144S | Missense | VUS | DM | LOVD | Likely Pathogenic |
| 46. | P186L | Missense | VUS | DM | LOVD | Likely Pathogenic |
| 47. | R218W | Missense | Pathogenic | DM | LOVD/ClinGen | Pathogenic |
| 48. | R218W | Missense | Pathogenic | DM | LOVD/ClinGen | Pathogenic |
| 49. | R218W | Missense | Pathogenic | DM | LOVD/ClinGen | Pathogenic |
| 50. | R218W | Missense | Pathogenic | DM | LOVD/ClinGen | Pathogenic |
| 51. | R218Q | Missense (hotspot) | Pathogenic | DM | LOVD/ClinGen | Pathogenic |
| 52. | G300V | Missense | Likely Pathogenic | DM | LOVD | Likely Pathogenic |
| 53. | G300V | Missense | Likely Pathogenic | DM | LOVD | Likely Pathogenic |
| 54. | G300V | Missense | Likely Pathogenic | DM | LOVD | Likely Pathogenic |
| 55. | V302M | Missense | Likely Pathogenic | DM | LOVD | Likely Pathogenic |
| 56. | E303K | Missense | Likely Pathogenic | DM | LOVD | Likely Pathogenic |
| 57. | R218W | Missense | Pathogenic | DM | LOVD/ClinGen | Pathogenic |
| 58. | T305A | Missense | VUS | DM | LOVD | Likely Pathogenic |
| 59. | R218W | Missense | Pathogenic | DM | LOVD/ClinGen | Pathogenic |
| 60. | R67W | Missense | VUS / Likely Pathogenic | DM | LOVD | Likely Pathogenic |
| 61. | R189S | Missense | VUS | DM | LOVD | Likely Pathogenic |
| 62. | R189S | Missense | VUS | DM | LOVD | Likely Pathogenic |
| 63. | M307I | Missense | Likely Pathogenic | DM | LOVD | Likely Pathogenic |
| 64. | M307I | Missense | Likely Pathogenic | DM | LOVD | Likely Pathogenic |
| 65. | P186T | Missense | VUS | DM | LOVD | Likely Pathogenic |
| 66. | P186T | Missense | VUS | DM | LOVD | Likely Pathogenic |
| 67. | G215D | Missense | Pathogenic | DM | LOVD | Pathogenic |
| 68. | C154F | Missense | Pathogenic | DM | LOVD | Pathogenic |
| 69. | T305P | Missense | VUS | DM | LOVD | Likely Pathogenic |
| 70. | R218W | Missense | Pathogenic | DM | LOVD/ClinGen | Pathogenic |
| 71. | Y68D | Missense | VUS | Reported | OMIM cited | Likely Pathogenic |
| 72. | R67W | Missense | VUS / Likely Pathogenic | DM | LOVD | Likely Pathogenic |
| 73. | G300D | Missense | VUS | DM | LOVD | Likely Pathogenic |
| 74. | R67W | Missense | VUS / Likely Pathogenic | DM | LOVD | Likely Pathogenic |
| 75. | R67W | Missense | VUS / Likely Pathogenic | DM | LOVD | Likely Pathogenic |
| 76. | D71N | Missense | Pathogenic / VUS (conflicting) | DM | LOVD | Likely Pathogenic |
| 77. | T75R | Missense | Likely Pathogenic | DM | LOVD | Likely Pathogenic |
| 78. | G146D | Missense | VUS | DM | LOVD | Likely Pathogenic |
| 79. | R189I | Missense | VUS | DM | LOVD | Likely Pathogenic |
| 80. | G300D | Missense | VUS | DM | LOVD | Likely Pathogenic |
| 81. | T75A | Missense | VUS | DM | LOVD | Likely Pathogenic |
| 82. | T75A | Missense | VUS | DM | LOVD | Likely Pathogenic |
| 83. | T75A | Missense | VUS | DM | LOVD | Likely Pathogenic |
| 84. | T75A | Missense | VUS | DM | LOVD | Likely Pathogenic |
| 85. | T75A | Missense | VUS | DM | LOVD | Likely Pathogenic |
| 86. | ΔFQ163-164 | In-frame deletion | Likely Pathogenic | DM | LOVD | Likely Pathogenic |
| 87. | ΔFQ163-164 | In-frame deletion | Likely Pathogenic | DM | LOVD | Likely Pathogenic |
| 88. | R67Q | Missense | VUS | DM | LOVD | Likely Pathogenic |
| 89. | T75M | Missense | VUS | DM | LOVD | Likely Pathogenic |
| 90. | R82W | Missense | Pathogenic | DM | LOVD | Pathogenic |
| 91. | T305A | Missense | VUS | DM | LOVD | Likely Pathogenic |
| 92. | T75M | Missense | VUS | DM | LOVD | Likely Pathogenic |
| 93. | T75M | Missense | VUS | DM | LOVD | Likely Pathogenic |
| 94. | R218W | Missense | Pathogenic | DM | LOVD/ClinGen | Pathogenic |
| 95. | T75R | Missense | Likely Pathogenic | DM | LOVD | Likely Pathogenic |
| 96. | R218Q | Missense (hotspot) | Pathogenic | DM | LOVD/ClinGen | Pathogenic |
| 97. | R218Q | Missense (hotspot) | Pathogenic | DM | LOVD/ClinGen | Pathogenic |
| 98. | W322C | Missense | Likely Pathogenic | DM | LOVD | Likely Pathogenic |
| 99. | W322C | Missense | Likely Pathogenic | DM | LOVD | Likely Pathogenic |
| 100. | P145L | Missense | Pathogenic | DM | LOVD | Pathogenic |
| 101. | R82Q | Missense | Pathogenic | DM | LOVD | Pathogenic |
| 102. | R82Q | Missense | Pathogenic | DM | LOVD | Pathogenic |
| 103. | R82Q | Missense | Pathogenic | DM | LOVD | Pathogenic |
| 104. | p.Ala91_Leu94del | In-frame deletion | Likely Pathogenic | DM | LOVD | Likely Pathogenic |
| 105. | p.Ala91_Leu94del | In-frame deletion | Likely Pathogenic | DM | LOVD | Likely Pathogenic |
| 106. | p.Ala91_Leu94del | In-frame deletion | Likely Pathogenic | DM | LOVD | Likely Pathogenic |
| 107. | G144D | Missense | VUS | DM | LOVD | Likely Pathogenic |
| 108. | G215D | Missense | Pathogenic | DM | LOVD | Pathogenic |
| 109. | G215D | Missense | Pathogenic | DM | LOVD | Pathogenic |
| 110. | G215D | Missense | Pathogenic | DM | LOVD | Pathogenic |
| 111. | 0 | — | NA | NA | Clinical ATS (no variant) | Clinical ATS diagnosis |
| 112. | C154F | Missense | Pathogenic | DM | LOVD | Pathogenic |
| 113. | T309I | Missense | VUS | DM | LOVD | Likely Pathogenic |
| 114. | G300D | Missense | VUS | DM | LOVD | Likely Pathogenic |
| 115. | N190I | Missense | VUS | DM | LOVD | Likely Pathogenic |
| 116. | T192A | Missense | VUS | DM | LOVD | Likely Pathogenic |
| 117. | T192A | Missense | VUS | DM | LOVD | Likely Pathogenic |
| 118. | T192I | Missense | VUS | DM | LOVD | Likely Pathogenic |
| 119. | R218Q | Missense (hotspot) | Pathogenic | DM | LOVD/ClinGen | Pathogenic |
| 120. | M307I | Missense | Likely Pathogenic | DM | LOVD | Likely Pathogenic |
| 121. | R218W | Missense | Pathogenic | DM | LOVD/ClinGen | Pathogenic |
| 122. | R218W | Missense | Pathogenic | DM | LOVD/ClinGen | Pathogenic |
| 123. | R67W | Missense | VUS / Likely Pathogenic | DM | LOVD | Likely Pathogenic |
| 124. | R218L | Missense | VUS | DM | LOVD | Likely Pathogenic |
| 125. | R218W | Missense | Pathogenic | DM | LOVD/ClinGen | Pathogenic |
| 126. | KCNJ2 Pos | — | Pathogenic (variant not specified) | DM/Reported | Reported positive | Pathogenic (reported) |
| 127. | R218W | Missense | Pathogenic | DM | LOVD/ClinGen | Pathogenic |
| 128. | R218W | Missense | Pathogenic | DM | LOVD/ClinGen | Pathogenic |
| 129. | p.75_76insThr | In-frame insertion | Likely Pathogenic | DM | LOVD | Likely Pathogenic |
| 130. | p.75_76insThr | In-frame insertion | Likely Pathogenic | DM | LOVD | Likely Pathogenic |
| 131. | p.75_76insThr | In-frame insertion | Likely Pathogenic | DM | LOVD | Likely Pathogenic |
| 132. | R67W | Missense | VUS / Likely Pathogenic | DM | LOVD | Likely Pathogenic |
| 133. | p.Glu153Asnfs*48 | Frameshift (nonsense) | Pathogenic | DM | LOVD | Pathogenic |
| 134. | R218W | Missense | Pathogenic | DM | LOVD/ClinGen | Pathogenic |
| 135. | R218W | Missense | Pathogenic | DM | LOVD/ClinGen | Pathogenic |
| 136. | — | — | NA | NA | Clinical ATS (no variant) | Clinical ATS diagnosis |
| 137. | — | — | NA | NA | Clinical ATS (no variant) | Clinical ATS diagnosis |
| 138. | V302M | Missense | Likely Pathogenic | DM | LOVD | Likely Pathogenic |
| 139. | R67Q | Missense | VUS | DM | LOVD | Likely Pathogenic |
| 140. | D71N | Missense | Pathogenic / VUS (conflicting) | DM | LOVD | Likely Pathogenic |
| 141. | — | — | NA | NA | Clinical ATS (no variant) | Clinical ATS diagnosis |
| 142. | T75M | Missense | VUS | DM | LOVD | Likely Pathogenic |
| 143. | T309I | Missense | VUS | DM | LOVD | Likely Pathogenic |
| 144. | R82W | Missense | Pathogenic | DM | LOVD | Pathogenic |
| 145. | R82Q | Missense | Pathogenic | DM | LOVD | Pathogenic |
| 146. | S136F | Missense | Pathogenic | DM | LOVD | Pathogenic |
| 147. | R218W | Missense | Pathogenic | DM | LOVD/ClinGen | Pathogenic |
| 148. | R218W | Missense | Pathogenic | DM | LOVD/ClinGen | Pathogenic |
| 149. | c.95_98del | — | — | — | — | — |
| 150. | p.Ala91_Leu94del | In-frame deletion | Likely Pathogenic | DM | LOVD | Likely Pathogenic |
| 151. | R218W | Missense | Pathogenic | DM | LOVD/ClinGen | Pathogenic |
| 152. | R218W | Missense | Pathogenic | DM | LOVD/ClinGen | Pathogenic |
| 153. | Y145C | Missense | Pathogenic | DM | LOVD | Pathogenic |
| 154. | Y145C | Missense | Pathogenic | DM | LOVD | Pathogenic |
| 155. | R218W | Missense | Pathogenic | DM | LOVD/ClinGen | Pathogenic |
| 156. | C101R | Missense | Likely Pathogenic | DM | LOVD | Likely Pathogenic |
| 157. | R218W | Missense | Pathogenic | DM | LOVD/ClinGen | Pathogenic |
| 158. | G144A | Missense | VUS | DM | LOVD | Likely Pathogenic |
| 159. | del 314-315 | In-frame deletion | Pathogenic | DM | LOVD | Pathogenic |
| 160. | R218W | Missense | Pathogenic | DM | LOVD/ClinGen | Pathogenic |
| 161. | D78Y | Missense | VUS | Reported | Case reports | Likely Pathogenic |
| 162. | T142P | Missense | Pathogenic (functional) | DM | LOVD | Pathogenic |
| 163. | A304D | Missense | VUS | DM | LOVD | Likely Pathogenic |
| 164. | D71Y | Missense | VUS | Reported | Case reports | Likely Pathogenic |
| 165. | — | — | NA | NA | Clinical ATS (no variant) | Clinical ATS diagnosis |
| 166. | — | — | NA | NA | Clinical ATS (no variant) | Clinical ATS diagnosis |
| 167. | G144A | Missense | VUS | DM | LOVD | Likely Pathogenic |
| 168. | G144A | Missense | VUS | DM | LOVD | Likely Pathogenic |
| 169. | G144A | Missense | VUS | DM | LOVD | Likely Pathogenic |
| 170. | G144A | Missense | VUS | DM | LOVD | Likely Pathogenic |
| 171. | G144A | Missense | VUS | DM | LOVD | Likely Pathogenic |
| 172. | G144A | Missense | VUS | DM | LOVD | Likely Pathogenic |
| 173. | G144A | Missense | VUS | DM | LOVD | Likely Pathogenic |
| 174. | E138K | Missense | Pathogenic | DM | LOVD | Pathogenic |
| 175. | M307I | Missense | Likely Pathogenic | DM | LOVD | Likely Pathogenic |
| 176. | C122Ter | Nonsense | Pathogenic | DM | LOVD | Pathogenic |
| 177. | M307V | Missense | VUS | DM | Reported | Likely Pathogenic |
| 178. | V302del | In-frame deletion | Pathogenic | DM | LOVD | Pathogenic |
| 179. | R218W | Missense | Pathogenic | DM | LOVD/ClinGen | Pathogenic |
| 180. | V200M | Missense | Likely Pathogenic | DM | ClinVar Likely Pathogenic | Likely Pathogenic |
| 181. | T305I | Missense | VUS | DM | Case reports | Likely Pathogenic |
| 182. | R218W | Missense | Pathogenic | DM | LOVD/ClinGen | Pathogenic |
| 183. | D78Y | Missense | VUS | Reported | Case reports | Likely Pathogenic |
| 184. | M307V | Missense | VUS | DM | Reported | Likely Pathogenic |
| 185. | Y145C | Missense | Pathogenic | DM | LOVD | Pathogenic |
| 186. | T192I | Missense | VUS | DM | LOVD | Likely Pathogenic |
| 187. | T192I | Missense | VUS | DM | LOVD | Likely Pathogenic |
| 188. | Y145C | Missense | Pathogenic | DM | LOVD | Pathogenic |
| 189. | T192I | Missense | VUS | DM | LOVD | Likely Pathogenic |
| 190. | G144A | Missense | VUS | DM | LOVD | Likely Pathogenic |
| 191. | R228ins | In-frame insertion | Likely Pathogenic | DM | LOVD | Likely Pathogenic |
| 192. | G300A | Missense | VUS | DM | Reported | Likely Pathogenic |
| 193. | KCNJ2 del | Large deletion | Pathogenic | Reported | OMIM | Pathogenic |
| 194. | KCNJ2 Neg | — | NA | NA | Clinical ATS (no variant) | Clinical ATS diagnosis |
| 195. | KCNJ2 Neg | — | NA | NA | Clinical ATS (no variant) | Clinical ATS diagnosis |
| 196. | N318S | Missense | VUS | DM | Reported | Likely Pathogenic |
| 197. | R312C | Missense | Pathogenic | DM | LOVD | Pathogenic |
| 198. | R218W | Missense | Pathogenic | DM | LOVD/ClinGen | Pathogenic |
| 199. | R218W | Missense | Pathogenic | DM | LOVD/ClinGen | Pathogenic |
| 200. | N216H | Missense | Pathogenic | DM | LOVD | Pathogenic |
| 201. | T305S | Missense | Likely Pathogenic | DM | Reported | Likely Pathogenic |
| 202. | T75M | Missense | VUS | DM | LOVD | Likely Pathogenic |
| 203. | T309I | Missense | VUS | DM | LOVD | Likely Pathogenic |
| 204. | — | — | NA | NA | Clinical ATS (no variant) | Clinical ATS diagnosis |
| 205. | G215D | Missense | Pathogenic | DM | LOVD | Pathogenic |
| 206. | R218W | Missense | Pathogenic | DM | LOVD/ClinGen | Pathogenic |
| 207. | T400M | Missense | VUS | DM | Reported | Likely Pathogenic |
| 208. | R218W | Missense | Pathogenic | DM | LOVD/ClinGen | Pathogenic |
| 209. | R218W | Missense | Pathogenic | DM | LOVD/ClinGen | Pathogenic |
| 210. | R218W | Missense | Pathogenic | DM | LOVD/ClinGen | Pathogenic |
| 211. | R218W | Missense | Pathogenic | DM | LOVD/ClinGen | Pathogenic |
| 212. | A304D | Missense | VUS | DM | LOVD | Likely Pathogenic |
| 213. | T309I | Missense | VUS | DM | LOVD | Likely Pathogenic |
| 214. | R218W | Missense | Pathogenic | DM | LOVD/ClinGen | Pathogenic |
| 215. | T192I | Missense | VUS | DM | LOVD | Likely Pathogenic |
| 216. | R82W | Missense | Pathogenic | DM | LOVD | Pathogenic |
| 217. | R67Q | Missense | VUS | DM | LOVD | Likely Pathogenic |
| 218. | p.Ala91_Leu94del | In-frame deletion | Likely Pathogenic | DM | LOVD | Likely Pathogenic |
| 219. | G146R | Missense (pore-loop) | Pathogenic | DM | LOVD | Pathogenic |
| 220. | G146R | Missense (pore-loop) | Pathogenic | DM | LOVD | Pathogenic |
| 221. | G300D | Missense | VUS | DM | LOVD | Likely Pathogenic |
| 222. | R312C | Missense | Pathogenic | DM | LOVD | Pathogenic |
| 223. | Y68D | Missense | VUS | Reported | OMIM cited | Likely Pathogenic |
| 224. | L222S | Missense | VUS | Reported | Case reports | Likely Pathogenic |
| 225. | L22S | Missense | Likely Benign | Not DM | ClinVar LB | Likely Benign |
| 226. | T75M | Missense | VUS | DM | LOVD | Likely Pathogenic |
| 227. | KCNJ2 Pos | — | Pathogenic (variant not specified) | DM/Reported | Reported positive | Pathogenic (reported) |
| 228. | E293K | Missense | VUS | DM | Reported | Likely Pathogenic |
| 229. | R312H | Missense | Pathogenic | DM | LOVD | Pathogenic |
| 230. | R40X | Nonsense | Pathogenic | DM | LOVD | Pathogenic |
| 231. | KCNJ2 Pos | — | Pathogenic (variant not specified) | DM/Reported | Reported positive | Pathogenic (reported) |
| 232. | T305I | Missense | VUS | DM | Case reports | Likely Pathogenic |
| 233. | KCNJ2 Pos | — | Pathogenic (variant not specified) | DM/Reported | Reported positive | Pathogenic (reported) |
| 234. | — | — | NA | NA | Clinical ATS (no variant) | Clinical ATS diagnosis |
| 235. | KCNJ2 Pos | — | Pathogenic (variant not specified) | DM/Reported | Reported positive | Pathogenic (reported) |
| 236. | P186R | Missense | VUS | DM | Reported | Likely Pathogenic |
| 237. | P186R | Missense | VUS | DM | Reported | Likely Pathogenic |
| 238. | R218Q | Missense (hotspot) | Pathogenic | DM | LOVD/ClinGen | Pathogenic |
| 239. | R218W | Missense | Pathogenic | DM | LOVD/ClinGen | Pathogenic |
| 240. | KCNJ2 Pos | — | Pathogenic (variant not specified) | DM/Reported | Reported positive | Pathogenic (reported) |
| 241. | KCNJ2 Pos | — | Pathogenic (variant not specified) | DM/Reported | Reported positive | Pathogenic (reported) |
| 242. | T74A | Missense | VUS | DM | LOVD/Reports | Likely Pathogenic |
| 243. | R218W | Missense | Pathogenic | DM | LOVD/ClinGen | Pathogenic |
| 244. | R218W | Missense | Pathogenic | DM | LOVD/ClinGen | Pathogenic |
| 245. | KCNJ2 pos | — | Pathogenic (variant not specified) | DM/Reported | Reported positive | Pathogenic (reported) |
| 246. | KCNJ2 Pos | — | Pathogenic (variant not specified) | DM/Reported | Reported positive | Pathogenic (reported) |
| 247. | R82W | Missense | Pathogenic | DM | LOVD | Pathogenic |
| 248. | — | — | NA | NA | Clinical ATS (no variant) | Clinical ATS diagnosis |
| 249. | R218W | Missense | Pathogenic | DM | LOVD/ClinGen | Pathogenic |
| 250. | R82W | Missense | Pathogenic | DM | LOVD | Pathogenic |
| 251. | P186Q | Missense | VUS | Reported | Case report only | Uncertain Significance |
| 252. | R218W | Missense | Pathogenic | DM | LOVD/ClinGen | Pathogenic |
| 253. | KCNJ2 pos | — | Pathogenic (variant not specified) | DM/Reported | Reported positive | Pathogenic (reported) |
| 254. | R218W | Missense | Pathogenic | DM | LOVD/ClinGen | Pathogenic |
| 255. | R82W | Missense | Pathogenic | DM | LOVD | Pathogenic |
| 256. | G215D | Missense | Pathogenic | DM | LOVD | Pathogenic |
| 257. | C122Y | Missense | VUS | DM | Reported | Likely Pathogenic |
| 258. | KCNJ2 del | Large deletion | Pathogenic | Reported | OMIM | Pathogenic |
| 259. | KCNJ2 pos | — | Pathogenic (variant not specified) | DM/Reported | Reported positive | Pathogenic (reported) |
| VUS = Variant of Uncertain Significance  DM = Disease Mutation  LOVD = Leiden Open Variation Database. | | | | | | |
