# Supplementary material for: Gender-specific cardiac features in Andersen–Tawil syndrome: a comprehensive meta-analysis of case reports and series
Source: J Interv Card Electrophysiol. 2026 Jan 14;69(6):1241–52. doi: 10.1007/s10840-026-02237-6 (PMC13645902; doi:10.1007/s10840-026-02237-6)
Supplement: Supplementary file 2 [file 10840_2026_2237_MOESM2_ESM.docx]

Supplementary Table 1

| Article | Sex | KCNJ2 mutation |
| --- | --- | --- |
| ALKHALFAN ET AL., 2022 | Female | KCNJ2 Pos |
| ALRASHED ET AL., 2019 | Female | E138K |
|  | Male | M3071 |
|  | Female | C122Ter |
|  | Female | M307V |
| ANDRADE ET AL., 2014 | Female | R218W |
| PALACIOS ARGUETA ET AL., 2017 | Female | KCNJ2 del |
| BARRÓN-DÍAZ ET AL., 2021 | Female | R218W |
|  | Female | S136F |
|  | Female | R218W |
|  | Female | R218W |
| BAUCH & KROGMANN, 2015 | Female | R218W |
| BENDAHHOU ET AL., 2005, 2007 | Male | C154F |
|  | Female | T305P |
|  | Male | C154F |
|  | Male | T3091 |
|  | Female | G300D |
|  | Male | T3091 |
| BIGELOW ET AL., 2015 | Female | c.95_98del |
|  | Female | D78Y |
| BÖKENKAMP ET AL., 2007 | Female | R218W |
|  | Male | R218W |
|  | Male | R218W |
| WÓJCIK-BOROWSKA ET AL., 2018 | Male | A304D |
|  | Male | A304D |
| ALLEN ET AL., 2020 | Male | L22S |
|  | Female | T75M |
| BURAKGAZI, 2019 | Male |  |
| CHAKRABORTY ET AL., 2015 | Male | R218W |
| CHAN ET AL., 2010 | Female | T1921 |
| CHOI ET AL., 2007 | Female | R218Q |
|  | Male | M3071 |
| CHUN ET AL., 2004 | Female | R67W |
|  | Female | C101R |
| DAVIES ET AL., 2005 | Female | R67W |
|  | Female | G300D |
|  | Female | G300D |
| DEEB ET AL., 2018 | Female | T1921 |
|  | Female | T1921 |
| DÉRI ET AL., 2021 | Female | E293K |
| DÍAZ-MANERA ET AL., 2014 | Male | G300A |
| DIKER ET AL., 2018 | Female | M307V |
| DONALDSON ET AL., 2003 | Female | R312C |
|  | Male | R67W |
|  | Female | D71N |
|  | Female | G146D |
|  | Female | R1891 |
|  | Female | G300D |
|  | Female | R312C |
|  | Male | R67W |
|  | Female | T75R |
| ECKHARDT ET AL., 2007 | Female | R67Q |
|  | Female | R82W |
|  | Female | T305A |
| EFREMIDIS ET AL., 2006 | Female |  |
| ERDOGAN ET AL., 2008 | Female |  |
| ERGÜL, 2018 | Female | V200M |
| FADAHUNSI ET AL., 2015 | Female | S136F |
| FADILAH ET AL., 2021 | Male | R312H |
| FERNÁNDEZ ET AL., 2018 | Female | R218W |
|  | Female | T3051 |
| FERNLUND ET AL., 2013 | Male | p.Ala91_Leu94del |
|  | Female | p.Ala91_Leu94del |
|  | Female | p.Ala91_Leu94del |
|  | Female | p.Ala91_Leu94del |
|  | Male | p.Ala91_Leu94del |
|  | Male | p.Ala91_Leu94del |
|  | Male | p.Ala91_Leu94del |
|  | Male | p.Ala91_Leu94del |
| FODSTAD, 2004 | Female | T75A |
|  | Male | T75A |
|  | Male | T75A |
|  | Male | T75A |
|  | Male | T75A |
|  | Female | AFQ163-164 |
|  | Female | AFQ163-164 |
| FRYER ET AL., 2015 | Female | R218W |
| FUKUMURA ET AL., 2019 | Female | G144A |
| GARCIA-TOUCHARD ET AL., 2007 | Female | T75M |
| HARUNA ET AL., 2007 | Male | R218W |
|  | Female | T3091 |
|  | Female | R67W |
|  | Female | R67W |
|  | Female | R67W |
|  | Male | G300V |
|  | Female | G215D |
|  | Female | G300V |
|  | Female | R67Q |
|  | Female | G146S |
|  | Female | R218Q |
|  | Female | R218W |
|  | Female | R218W |
|  | Male | R218W |
|  | Male | R218W |
|  | Female | R218Q |
|  | Male | R218Q |
| HASEGAWA ET AL., 2015 | Male | 75_76insThr |
|  | Female | 75_76insThr |
|  | Female | 75_76insThr |
| HAYASHI ET AL., 2014 | Male | R67Q |
| INAGAKI ET AL., 2021 | Female | R40X |
| RANSOM ET AL., 2023 | Female | KCNJ2 Pos |
| JANSON ET AL., 2014 | Male | R218W |
|  | Female | R218W |
| PARSONNET, 2009 | Female | KCNJ2 pos |
| JUNKER ET AL., 2002 | Female | R218W |
| KAMATE & CHETAL, 2011 | Male |  |
| KANNANKERIL ET AL., 2004 | Female | KCNJ2 Pos |
| AIREY ET AL., 2009 | Female | KCNJ2 Neg |
| KHAN ET AL., 2018 | Male |  |
| KIM ET AL., 2016 | Female | G215D |
| KIMURA ET AL., 2012 | Female | R40X |
|  | Male | R218Q |
|  | Female | R218W |
|  | Male | R218W |
|  | Male | R80C |
|  | Female | R82Q |
|  | Female | R82W |
|  | Female | R218W |
|  | Male | R218W |
|  | Male | R228ins |
|  | Male | R82W |
| KOKUBUN ET AL., 2019 | Female | R67W |
|  | Male | R67Q |
| KOSTERA-PRUSZCZYK ET AL., 2015 | Male | T75M |
|  | Female | T3091 |
|  | Female | R82W |
|  | Female | R82Q |
|  | Female | T75M |
|  | Male | T3091 |
|  | Female | G146R |
|  | Female | G146R |
| KRYCH ET AL., 2019 | Female | Glu153Asnfs*48 |
| KURAMOTO ET AL., 2012 | Female | L382L |
| LE TANNO ET AL., 2021 | Female | R82W |
| LIM ET AL., 2010 | Female | P186Q |
|  | Male | G144D |
|  | Male | G215D |
|  | Female | G215D |
|  | Female | G215D |
| LIMBERG ET AL., 2013 | Male | R218Q |
|  | Male | R218Q |
|  | Female | W322C |
|  | Female | W322C |
|  | Female | P145L |
|  | Female | R82Q |
|  | Male | R82Q |
|  | Female | R82Q |
|  | Female | N318S |
| LU, 2006 | Female | T75R |
| MAFFÈ ET AL., 2020 | Male | T1921 |
|  | Female | T1921 |
| MÁRQUEZ ET AL., 2015 | Female | G144A |
| MARQUIS-NICHOLSON ET AL., 2014 | Female | KCNJ2 del |
| MARRUS ET AL., 2011 | Male | D71Y |
| NAGASE ET AL., 2007 | Female | T192A |
| NAGASHIMA ET AL., 2010 | Female | T192A |
| NAGPAL ET AL., 2023 | Female | KCNJ2 Pos |
| NGUYEN & FERNS, 2018 | Female | D71N |
| NOZAKI ET AL., 2016 | Male | R218W |
| OBEYESEKERE ET AL., 2011 | Female | T400M |
| OLIVEIRA ET AL., 2018 | Female | KCNJ2 pos |
| ONORE ET AL., 2024 | Female | R218W |
|  | Male | R218W |
| ÖRDÖG ET AL., 2015 | Female | V302del |
| ORETO ET AL., 2023 | Male | T3051 |
| PARK ET AL., 2023 | Female | KCNJ2 Pos |
| PATEL ET AL., 2023 | Male |  |
|  | Male |  |
| PELLIZZÓN ET AL., 2008 | Female | R67W |
| PETERS ET AL., 2007 | Male | KCNJ2 Neg |
| PYO ET AL., 2013 | Female | KCNJ2 Neg |
| RAI ET AL., 2019 | Female | G144A |
|  | Female | G144A |
|  | Female | G144A |
|  | Female | G144A |
|  | Male | G144A |
|  | Male | G144A |
|  | Female | G144A |
| RAJAKULENDRAN ET AL., 2010 | Female | R218W |
|  | Female | R218W |
| P ET AL., 2015 | Male |  |
| RANSOM ET AL., 2023B | Female | KCNJ2 Pos |
| REZAZADEH ET AL., 2016 | Male | L222S |
| RUJIRACHUN ET AL., 2021 | Female | P186R |
|  | Female | P186R |
| SAMANTA, 2019 | Female | R218W |
| SANSONE ET AL., 1997 | Male | R218W |
| SATO ET AL., 2019 | Female | R228ins |
| SCAVEE ET AL., 2018 | Female | C122Y |
| SCHEIPER ET AL., 2017 | Female | Y145C |
|  | Male | Y145C |
| SCHOONDERWOERD ET AL., 2006 | Male | R218W |
|  | Female | R218W |
|  | Female | R218W |
| SHANDLING & SAFANI, 2008 | Female |  |
| SHIBUYA ET AL., 2019 | Male | V302M |
| SHIMIZU & HORIE, 2011 | Female | R67W |
| ŠINKOVEC ET AL., S. F. | Female | T142P |
| SONG ET AL., 2016 | Female | R218W |
|  | Male | T305A |
|  | Male | R218W |
|  | Male | R67W |
|  | Male | R189S |
|  | Male | R189S |
|  | Female | M3071 |
|  | Female | M3071 |
|  | Female | P186T |
|  | Female | P186T |
|  | Female | G215D |
|  | Female | R218W |
| SUBBIAH ET AL., 2008 | Female | R218W |
| SUMITOMO ET AL., 2008 | Female | N1901 |
| TAKAHASHI ET AL., 2005 | Male | R218W |
| TAN ET AL., 2012 | Female | Y68D |
|  | Male | Y68D |
| TANI ET AL., 2007 | Female | T75M |
|  | Female | T75M |
| TENGAN ET AL., 2006 | Male | R218W |
| THAKKAR ET AL., 2012 | Male | - |
|  | Male | - |
| AI ET AL., 2002 | Male | T192A |
|  | Female | T192A |
| TRISTANI-FIROUZI ET AL., 2002 | Female | 95_98del |
|  | Female | S136F |
|  | Female | G144S |
|  | Male | P186L |
|  | Female | R218W |
|  | Male | R218W |
|  | Male | R218W |
|  | Male | R218Q |
|  | Female | G300V |
|  | Male | V302M |
|  | Female | E303K |
|  | Female | R218W |
|  | Female | N216H |
|  | Female | R218W |
| TULLY ET AL., 2015 | Female | Y145C |
|  | Female | Y145C |
|  | Female | R218W |
|  | Female | R218W |
|  | Female | R218W |
| TUNÇER ET AL., 2015 | Male | R218W |
| VAN ERT ET AL., 2017 | Female | R218L |
| WEIR ET AL., 2011 | Female | R218W |
| WANG ET AL., 2011 | Female | R82W |
|  | Female | R82W |
| WU, 2020 | Female | R218Q |
| YANG ET AL., 2022 | Female | R218W |
| YILDIZ ET AL., 2024 | Female | KCNJ2 pos |
| YILMAZ & KANAT, 2021 | Female | KCNJ2 Pos |
|  | Female | KCNJ2 Pos |
| YOON ET AL., 2006 | Male | G300V |
|  | Female | G300V |
|  | Female | R218W |
|  | Female | del 314-315 |
|  | Female | R218W |
|  | Female | D78Y |
| ZALESKA ET AL., 2018 | Female | KCNJ2 Pos |
| ZHAO ET AL., 2023 | Female | T74A |
